# Validation of the French version of the « Meta-Cognition Questionnaire » for adolescents (MCQ-Af): Evolution of metacognitive beliefs with age and their links with anxiety during adolescence

**DOI:** 10.1371/journal.pone.0230171

**Published:** 2020-03-11

**Authors:** Yasmina Lachat Shakeshaft, Thierry Lecerf, Larisa Morosan, Deborah Myriam Badoud, Martin Debbané

**Affiliations:** 1 Developmental Clinical Psychology Unit, Faculty of Psychology and Educational Science, University of Geneva, Geneva, Switzerland; 2 Differential Clinical Psychology Unit, Faculty of Psychology and Educational Science, University of Geneva, Geneva, Switzerland; 3 Research Department of Clinical, Educational and Health Psychology, University College London, London, United Kingdom; Chinese Academy of Medical Sciences and Peking Union Medical College, CHINA

## Abstract

**Introduction:**

The Meta-Cognitions Questionnaire for Adolescents (MCQ-A) measures individual differences of metacognitive beliefs and monitoring thought to be involved in the onset and maintenance of psychological disorders, especially in those involving anxiety. This assessment tool has been employed in research and clinical settings involving French-speaking adolescents, but appropriate validation has yet to be conducted. This article aims to first validate the francophone version of the MCQ-Af using measures sensitive to the expression of anxiety, and secondly, to examine the influence of age and gender on metacognitive beliefs, anxiety and their links.

**Method:**

214 adolescents (114 females) between 13 and 17 completed the MCQ-Af (French version) as well as the Revised-Children's Manifest Anxiety Scale (R-CMAS), French version, to assess anxiety manifestations. Structural validity was examined with confirmatory factor analyses. Three models were compared to the higher order five factor model proposed in the original validation study. Internal consistency and test-retest reliability were also performed. Student’s t tests as well as simple and stepwise regressions were conducted to assess for age and gender.

**Results:**

The five correlated factors retained in the original version of the MCQ were replicated, and confirmatory factor analyses yielded comparable fit indices for a covariate factor model, as well as for a bifactor model. The bifactor model was privileged for theoretical reasons. Analyses were performed on a shortened questionnaire of 27 items as 3 items (2, 12 and 14) had non-significant loadings in prior path analyses. Age and gender differences were found in specific sub-factors of the MCQ-Af; positive and negative metacognitive beliefs seem to increase with age; girls seem to score higher on the negative metacognitive beliefs and thought control dimensions. The study further reports specific links between anxiety manifestation and negative and positive metacognitive beliefs, as well as confidence in one’s memory. A modest influence of age and gender on this link was also highlighted.

**Conclusion:**

The present research provides the first evidence that the MCQ-Af is a valid and reliable instrument to assess individual differences of metacognitive beliefs in French-speaking adolescents. Nevertheless, it highlights that caution should be taken in regards of 3 items in particular (items 2, 12 and 14). Furthermore, age and gender in assessed samples of adolescents might influence the scores of the different dimensions of the questionnaire.

## Introduction

The Meta-Cognition Questionnaire (MCQ) was developed by Cartwright-Hatton and Wells [1] to quantify the quality of metacognitive beliefs and processes that are contributing to the elaboration and maintenance of emotional disorders. The questionnaire relates to different levels of metacognition, metacognitive beliefs and processes such as monitoring and control in link with worries and meta-worries that are central to the Metacognition Theory (MCT) developed by Wells and colleagues [2–4].

### The Metacognition Theory (MCT)

The MCT [2–4] considers three interactive levels of cognition where the lowest level relates to the automatic processing of external, affective, cognitive or physiological stimuli, the second level to the cognitive style or processes of thoughts and actions maintaining cognitive self-regulation in response to intrusions, and the higher level to the meta-system encompassing beliefs, plans and metacognitive knowledge stored in memory [2–4]. In psychologically vulnerable individuals, the second level of cognitive style is triggered by the lower level’s intrusive or misinterpreted stimuli. This intermediate level is continually sustained by the higher meta-system with the means of highly controlled and monitored thoughts, plans or beliefs. Wells and Matthews [5] in the Self-Regulatory Executive Function (S-REF) model stipulates that the cognitive style is represented as a persistent cycle where thoughts may be caught in a maladaptive style of appraisal and excessive self-centered attention, defined as the Cognitive Attentional Syndrome (CAS). This syndrome takes form in worries and ruminations, and is thought to function on negative information, directing attention towards threatening stimuli. Furthermore, such attentional strategies and excessive worries employed for self-regulation often fail to update the metacognitive knowledge of the higher level. Captured in this vicious circle, these excessive and disruptive worries can become the sole coping strategy used by an individual to react not only in face of threats but moreover to respond to emotions generated by the worries themselves. These worries about worries or meta-worries could then lead to psychological suffering and emotional distress [5].

### The metacognition questionnaire

The Meta-Cognition Questionnaire (MCQ) for adults was developed by Cartwright-Hatton and Wells in 1997 [1] to quantify the quality of metacognitive beliefs (higher level of metacognition) and processes (intermediate level of metacognition) described in the MCT. The MCQ-A was then adapted for adolescents in 2004 by Cartwright-Hatton, Mather, Illingworth, Brocki, Harrington et Wells [6]. The adult version was adapted and validated in many different languages [7–13], as well as the adolescent version [14–17] and the child version [18, 19]. Even though the MCQ French version for adult has been validated a few year ago [7], the need to use an adolescent version for our young French population in both clinical and non-clinical settings is still very much needed. Our aim is therefore to provide a valid questionnaire for the French-speaking adolescent population.

### Metacognitive beliefs and psychopathology

The Meta-Cognition Questionnaires for adults (MCQ) and Adolescents (MCQ-A) are widely used in clinical psychology and psychiatric research. Initially, the S-REF model and the CAS were described in Wells’ metacognitive model of generalized anxiety disorder (GAD). Worries associated with GAD that would be similar in nature and in content to the « normal and adaptive worries » are called « positive metacognitive worries » [20] i.e. item 7 of the MCQ « I need to worry in order to keep organized ». But worries particularly linked to the beliefs about the uncontrollability of thoughts and about worries themselves, are considered as maladaptive, e.g. « negative metacognitive worries and beliefs » [5]. When associated, those positive and negative metacognitive worries as represented in Well’s model can lead not only to GAD but also to other emotional and psychological disorders such as social anxiety [4], obsessive-compulsives disorder (OCD) [21], post-traumatic stress disorder (PTSD) [22] as well as depression [23] and psychotic spectrum disorder (PSD) [24].

In this study we will analyze the relationship between the MCQ-A French version and anxiety manifestations for the purpose of comparing the results with the original validation of the English adolescent questionnaire [6].

### Metacognitive beliefs and adolescence

Studies having measured metacognitive beliefs, as well as control and monitoring of thoughts using the MCQ, yield heterogeneous results concerning their evolution with age, in clinical or non-clinical samples [6, 25–28]. Some authors have suggested that metacognitive beliefs at the age of 13 could be compared to metacognitive beliefs in adulthood [25, 27, 29].

However, the literature converges on the developmental considerations, namely that a difficulty of thought control would only be conceptually valid around the age of nine [30] as the cognitive abilities needed to develop such metacognitive beliefs, especially those related to anxiety, would require the ability to predict the future and the ability to develop hypothetical consequences and solutions [31]. Thus, the development of metacognitive beliefs is gradual [32, 33] and requires increasing ability to control, plan and self-regulate behavior [31]. Moreover, developmental neuroscience demonstrates that a number of different higher order psychological processes, sustained by metacognitive beliefs, such as thinking about one own’s cognitive and emotional functioning, have particular maturation trajectories during adolescence until mid-to-late adolescence before showing relative stability [34–36]. Furthermore, Van Duijvenvoorde and al. (2016) observed age-related changes in prefrontal connectivity with subcortical structures implicated in emotional evaluation, control and monitoring [37]. It is also corroborated by studies that grey matter and white matter volumes have different trajectories in males and females [38].

As these studies suggest, behavioral and emotional self-regulation as well as metacognitive processes should continue to mature until late adolescence. These observations motivated the present inquiry to ask whether age and gender influence the expression of metacognitive beliefs in a sample of francophone adolescents.

### The present study

First, we test the structural validity of the scores’ interpretation of the Meta-Cognition Questionnaire for Adolescent, French version (MCQ-Af), with confirmatory factor analyses. In Cartwright and Wells’ first validation [6], the author concluded to a higher order factor model, meaning that all 5 factors would load on a single general factor illustrating the MCQ-A total score. However, numerous studies having used the MCQ in the context of different psychopathologies, have shown very different patterns of relations between the general factor with each of the five factors, the most influential being negative metacognitive beliefs for a great majority [4, 21–24]. We expect that the bifactor model would best explain the scores in our population. In this model, the common variances of items would be revealed in the so-called « breadth factor » rather than in the indirect loading of a « general higher order factor » on all items. Bifactor models are often used in cognitive and psychopathology research to distinguish the relative importance of domain specific factors over a general factor [39–41]. In our case it is indeed of particular interest because it will bring to the fore the independent variances of the five factors recognized as having dissimilar patterns of influence over other variables, such as psychopathological manifestations [4, 21–24].

We then complete the validation of the MCQ-Af, with the analyses of internal consistency (Cronbach’s α) and test-retest reliability (Pearson’s correlation r).

Our second aim is to investigate the links between MCQ-Af and Revised-Children’s Manifest Anxiety Scale (R-CMAS) [6, 42], compare the results to the ones of this original validation of the English version [6] and confirm this link with simple and stepwise regressions.

Finally, consistent with developmental psychology and neuroscience research, we hypothesize that age will impact the scores of this self-reported measure of metacognitive beliefs and processes. As the young people in our sample, aged between 13 and 17, have not fully developed the connectivity between pre-frontal and subcortical regions [37], their metacognitions would not have reached the same level as adults’ metacognitions. Therefore, we expect the scores of the MCQ-Af would continue to significantly change with age.

## Methods

### Participants

Initially, a total of 245 adolescents between 13 and 17 were enrolled to participate in this study. Advertisements addressed to headmasters were distributed in schools of French-speaking Switzerland and were approached by YLS who presented the study. The Meta-Cognition Questionnaire for Adolescents, French version (MCQ-Af) and the Revised-Children’s Manifest Anxiety Scale (R-CMAS), French version [42] were administered in the classroom, in the presence of their primary teacher and under the supervision of YLS. Written informed consent was received from all 245 participants and their parents under protocols approved by the cantonal ethics committee for human research of Geneva, Switzerland, and by the ethics committee of the psychology and educational sciences department of the university of Geneva.

From the 245 submitted MCQ-Af, we excluded 12 questionnaires (4 non-answered and 8 of non French-speaking participants), as well as 19 incomplete questionnaires. Therefore, 214 adolescents (100 boys, 114 girls between 13 and 17, average age 15.43 (SD = 1.25) were finally integrated in the validation analysis of the MCQ-Af.

A subsample of 64 students (26 boys, 38 girls, aged between 15 and 17, M = 15.76; SD = .84) answered the MCQ-Af a second time after an average time of 2.07 weeks (after 2 weeks for all classes, except after 3 weeks for 1 class of 15) for the purpose of test-retest analyses.

The analyses of the links between metacognition and anxiety were based on a sample of 197 adolescents (97 boys, 100 girls, between 13 and 17, mean age 15.28, SD = 1.21), incomplete questionnaires and questionnaires showing a score above the 90^th^ percentiles on the social desirability scale (n = 3) of the R-CMAS having been disregarded.

### Measures

**The MCQ-A** [6] was originally validated with a five factor solution, each factor consisting of 6 items rated on a 4-point Likert scale, ranging from « do not agree », « agree slightly », « agree moderately » and « agree very much ». For each factor the minimum score is therefore 6 and the maximum score 24, the minimum total score being 30 and the maximum total score 120. Factor 1: « Positive metacognitive beliefs » (factor MCpos). Factor 2: « Negative metacognitive beliefs » (factor MCneg). Factor 3: « Cognitive confidence » (factor Confidence). Factor 4: « Negative beliefs about thoughts in general linked to superstitions, punishment and responsibility » (factor Control). Factor 5: « Cognitive self-consciousness » or « monitoring » (factor Conscience). The items’ translation was reproduced from the French version of the adult MCQ (65 items) made by Laroi, Van der Linden et d’Acremont [7], who proceeded with a forward-backward translation. All 30 translated corresponding items were retained, with the exception of item 2 that was adapted to a more adolescent language. Of note, this item was also adapted from the English adult version to the corresponding adolescent version [6].

**The Revised-Children’s Manifest Anxiety Scale (R-CMAS), French version** [42] consists of 37 written questions answered by « yes » or « no ». The four subscales are physiological anxiety (somatic manifestations, 10 items), worries and sensitivity (vulnerability to environmental pressures, 11 items), social worries (worries about self in relation to others, 7 items) and a subscale of social desirability (9 items). The addition of the first 3 subscales (28 items) equals the total score of anxiety. The scores are standardized for children and adolescents between 6 and 19 and for gender (score T = 10, SD ± 3 for subscales; score T = 50, SD ± 10, for total score). High anxiety levels correspond to high scores, total score varying between 23 and 81, a score above 60 being considered of clinical interest. The questionnaire has good psychometric qualities, an internal consistency of α = .84, reliability correlations between .62 and .74 and convergent and construct validities of a relatively good sensitivity. However the physiological anxiety subscale index has a rather low sensitivity [43].

### Analysis procedure

The process of validation of the MCQ-Af included confirmatory factor analyses (CFA), measuring of internal consistency (Cronbach’s α) and reliability by test/re-test (Pearson’s correlation *r*). We also analyzed influence of age and gender with simple and stepwise linear regressions.

Analyses were performed using Statistica version 12. CFA was conducted with structural equation modelling (SEM) using SPSS-Amos, version 23. We proceeded by comparison of 4 models e.g. the unidimensional factor model, the second order factor model, the covariate factor model and the bifactor model. The unidimensional factor model (model 1) considers a single general factor loading on all items directly. The second order factor model (model 2) illustrates the loadings of a single higher order factor on all 5 factors as proposed in the original English validation [6]. The bifactor model (model 3) explains, on the one hand, a unique factor (the breadth factor) explaining the shared variance of all items, and, on the other hand, the 5 factors explaining specific variances. Finally, the covariate factor model (model 4) shows the covariances of the 5 factors without considering a higher order factor.

As the questionnaire is using a 4 Likert response scale, scores are generally considered categorical. Model fit was therefore estimated using the unweighted least squares (UWLS) as is appropriate when handling with categorical factor indicators and assuming violation of multivariate normality. Several fit indices were selected to evaluate model fit. First we examined the *χ2* depending on sample size being therefore rarely non-significant [44]. We therefore also examined the goodness of fit index (GFI) evaluating the correspondence between the model and the data, values above 0.90 indicating a good model fit [45]. Discrepancy of residual’s covariance matrices were estimated using Root Mean square Residual (RMR) which indicates a good fit when value is lower than 0.08 and an excellent fit when lower than 0.05 [44]. For baseline comparison, we selected the Normed Fit Index (NFI) showing how far between the independent and the saturated model the default model is situated (the default model improves the independent model by the obtained value percentage) and the relative Fit Index (RFI) independent of sample size; values of both indices greater than 0.90 and 0.95 respectively indicating a good fit [45–48]. Finally, for parsimony comparison, we also indicated the Parsimonious Normed Fit Index (PNFI) taking into account complexity of the models.

The link between metacognition and anxiety was first examined with the correlations of the MCQ-Af and the R-CMAS [42] scores. In their article Cartwright-Hatton and al. [6] have observed a significant link between factor MCneg and anxiety manifestations. We examined this link further with simple and stepwise regressions taking also into account the effect of age and gender.

## Results

All factor scores and the total score of the MCQ-Af were normally distributed. The score means of our sample of 214, aged 13 to 17 (100 boys, 114 girls, mean age 15.43, SD = 1.25) and subsample of 64, aged 15 to 17 (26 boys, 38 girls, mean age 15.76, SD = 0.84), were comparable but slightly higher than the one’s obtained by Cartwright-Hatton and al. [6] based on a sample of 166 English-speaking adolescents of the same age (Table 1).

**Table 1:** Comparison of means and standard deviations (SD) of the 5 factors of the MCQ-A and MCQ-A total score.

	C-H		
	N = 166	N = 214	*n* = 64
Age range	13–17	13–17	15–17
% girls	65.7%	53.3%	59.4%
MCQ-A	58.5 (15.0)	61.48 (12.01)	66.53 (7.71)
MCpos	10.7 (4.5)	11.29 (3.89)	12.25 (3.41)
MCneg	12.4 (4.8)	12.03 (3.81)	14.02 (3.46)
Confidence	10.3 (3.7)	10.73 (3.83)	10.91 (3.22)
Control	11.5 (3.6)	11.66 (3.67)	12.88 (3.82)
Conscience	13.9 (4.0)	15.75 (3.72)	16.43 (3.41)

Scores in Cartwright-Hatton and al. (C-H) N = 166 and our samples of N = 214 and *n* = 64.

**Individual Confirmatory Factor Analysis** (CFA) of items and their own factor showed that item 2, 12 and 14 were problematic as the loadings were non-significant. Overall loadings had the following values: standardized loadings between .49 et .77 for MCpos; between .43 et .78 for MCneg (except .06 for item 2) between .68 and .90 for Confidence (except for .32 for item 14); between .45 et .72 for Control; between .46 et .82 for Conscience (except for .18 for item 12). Items 2, 12 and 14 loading below the value 0.35 and non-significant were therefore excluded from our subsequent analysis.

**Fig 1:**
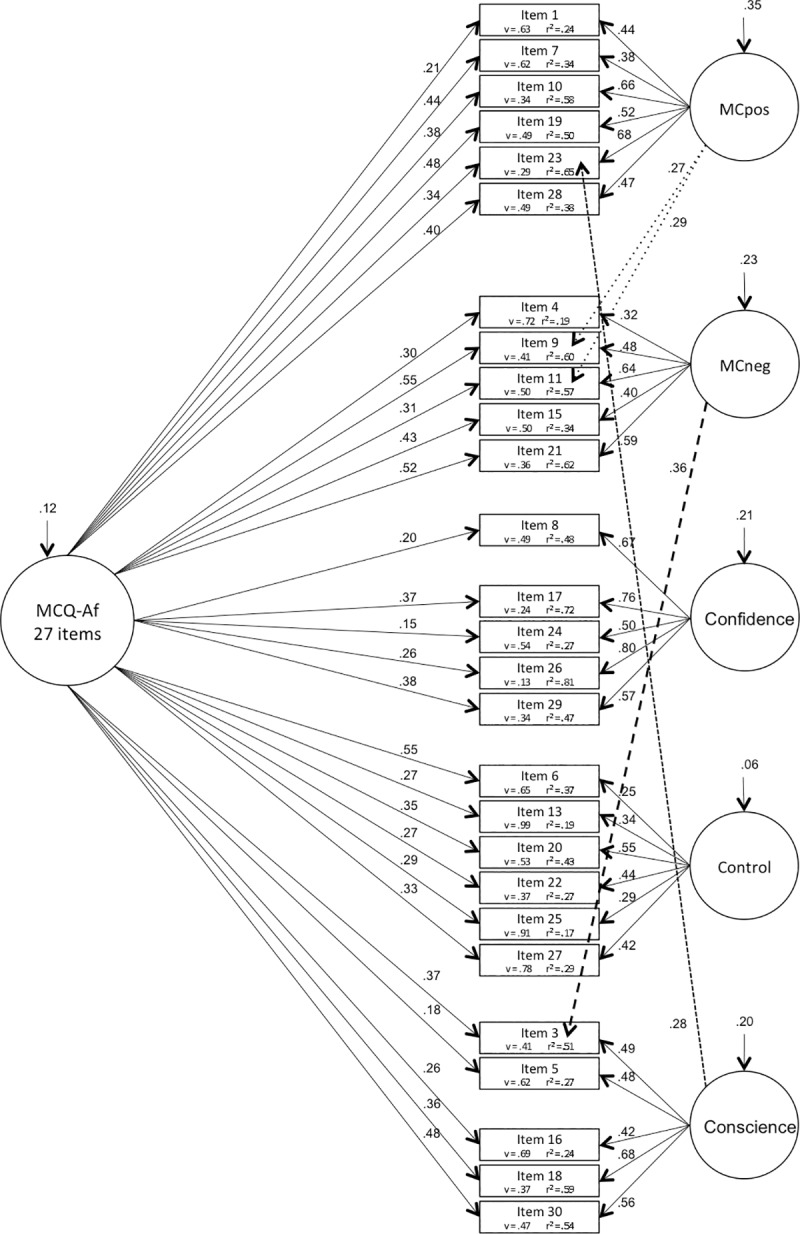
Bifactor model of MCQ-Af (27questions). Numbers arrowed on latent variables represent variance, v = variance; r^2^ = squared multiple correlations.

**Fig 2:**
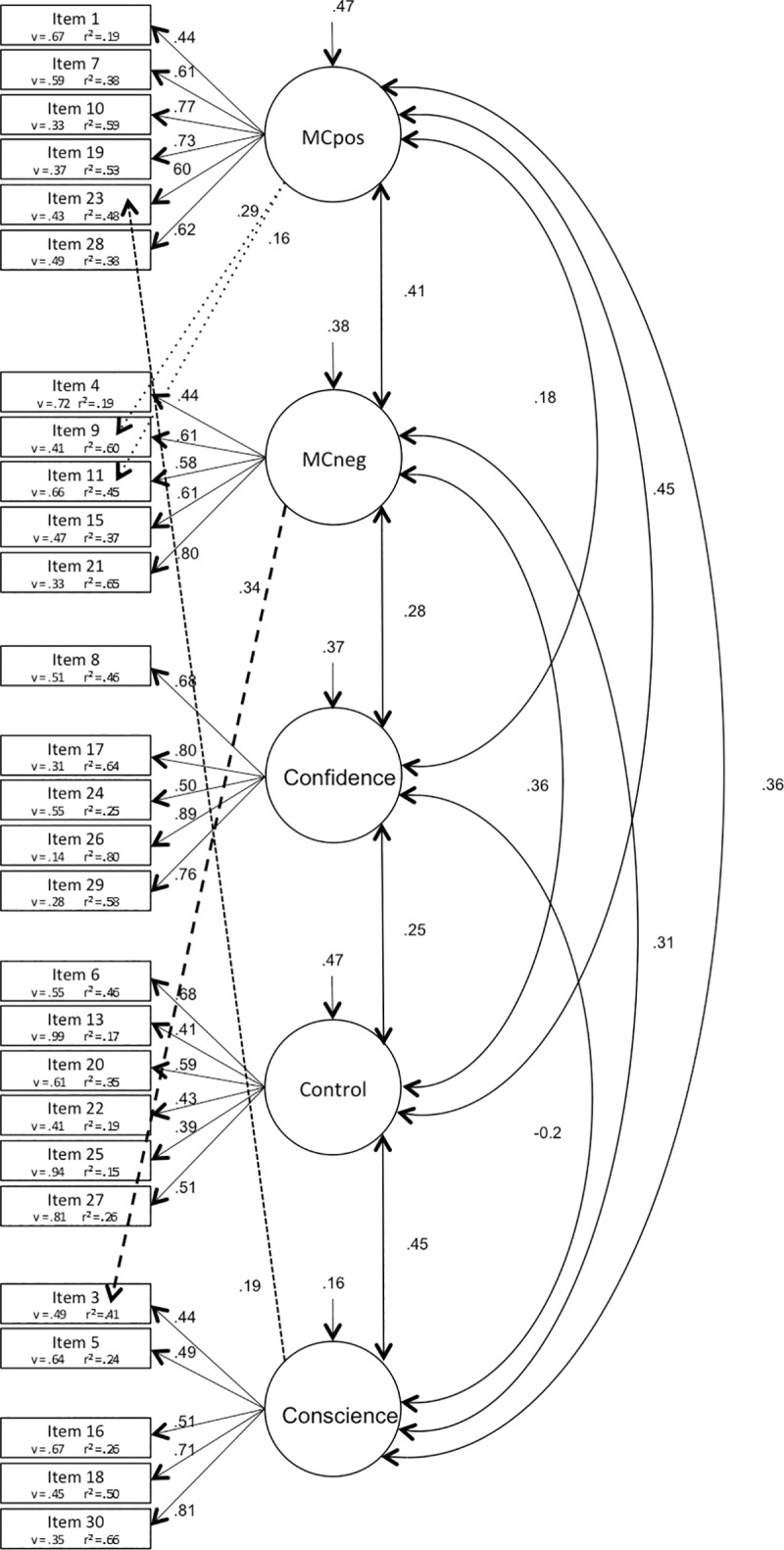
Covariate factor model of MCQ-Af (27questions). Numbers arrowed on latent variables represent variance, v = variance; r^2^ = squared multiple correlations.

**The CFA** was therefore completed with the shortened questionnaire comprising 27 items (MCQ-Af27). We first compared 4 different models, the unidimensional factor model (model 1), the second order factor model (model 2), the bifactor model (model 3) and the covariate factor model (model 4). Loadings of residuals on items were fixed to 1, as well as the minimum amount of loadings of latent variables on items i.e. one loading of each latent variable on a corresponding item. While the unidimensional factor model showed the worst fit indices, the bifactor model and the covariate factor model yielded the best indices. Nevertheless, the two latter had an RFI below 0.9 (Table 2). Modification indices revealed that allowing 4 items to load on multiple factors would improve the model fit. Therefore, we examined model 2, 3 and 4 with those 4 cross-loadings and noticed a better fit for each model 2a (the higher order model), 3a (the bifactor model, Fig 1) and 4a (the covariate factor model, Fig 2), see Table 2.

**Table 2:** Comparison of model fit indices of 4 models of the MCQ-Af27.

	χ^2^	df	RMR	GFI	NFI	RFI	PGFI
Model 1 Unidimensional	933,13	328	0,108	0,827	0,692	0,670	0.717
Model 2 Higher order	862,71	324	0,104	0,840	0,715	0,691	0.722
Model 2a	670,70	318	0,091	0,875	0,778	0,755	0.736
Model 3 Bifactor	334,30	301	0,064	0,938	0,890	0,871	0.747
Model 4 Covariate	287,44	314	0,060	0,947	0,905	0,894	0.786
Model 3a	271,03	297	0,058	0,950	0,910	0,894	0.752
Model 4a	248,48	310	0,056	0,954	0,918	0,907	0.782

Model 2a, model 3a and model 4a: respective models with cross loadings.

**Correlations** of the 5 factors of the shortened MCQ-Af27 questionnaire were positive and significant, indices varying between *r* = .14 and *r* = .41 (*p* < .001); the only non-significant correlation was found between factors Confidence and Conscience (*r* = .02, *p* = .808) (Table A in S1 Appendix). Power for Pearson’s correlation *r* = .20 considering our 214 participants was 84%, maximum power obtained for a correlation of *r =* .34.

### Internal consistency

Cronbach’s alphas were of acceptable values and comparable to Cartwright-Hatton and al. [6], particularly if considering the corrected MCQ-Af27, showing acceptable internal consistency for all 5 factors and total score, α between .684 and .852 (Table 3).

**Table 3:** Internal consistency and reliability of MCQ-A scores.

	Cronbach’s alpha		Reliability (Pearson *r*)		Mean T1	Mean T2	CI	%
	C-H	N = 214	C-H	*n* = 64	*n* = 64	*n* = 64	*n* = 64	*n* = 64
MCQ-A30	0.91	0.842	.34 (*p* = .017)	.77 (*p* < .001)	66.53 (7.71)	62.68 (9.29)	[63.44;69.62]	-
MCQ-Af27	-	0.852	-	.78**	58.49 (8.10)	54.85 (9.20)	[55.56;61.42]	68.75
MCpos	0.88	0.807	0.77	.73**	12.25 (3.41)	11.59 (3.67)	[10.81;13.69]	67.19
MCneg	0.84	0.726	0.24	.78**	14.02 (3.46)	13.64 (4.01)	[12.74;15.30]	-
MCneg-Q2	-	0.783	-	.75**	11.97 (3.43)	11.42 (3.8)	[10.56;13.38]	62.25
Confidence	0.81	0.811	0.8	.76**	10.91 (3.22)	11.01 (3.67)	[9.63;12.19]	-
Confidence-Q14	-	0.844	-	.71**	8.3 (3.28)	8.12 (3.02)	[6.93;9.67]	71.88
Control	0.66	0.684	0.9	.77**	12.88 (3.82)	11.50 (3.73)	[11.52;14.24]	70.31
Conscience	0.79	0.732	0.83	.69**	16.43 (3.41)	14.81 (3.61)	[14.84;18.02]	-
Conscience-Q12	-	0.754	-	.63**	13.69 (2.93)	12.30 (3.14)	[12.05;15.33]	81.25

Results in Cartwright-Hatton and al. (C-H) and our samples of N = 214 and *n* = 64; MCpos = positive metacognitive beliefs; MCneg = negative metacognitive beliefs. T1 = time of test. T2 = time of re-test. CI = 90% Confidence Interval. % = percentages of participants in the 90% Confidence Interval. In grey: results of MCQ-Af27 without item 2, 12 and 14 (Q2, Q12 and Q14).

** p < .001

### Test-retest reliability

Sixty-four participants (*n* = 64; 26 boys, 38 girls between 15 and 17, mean age 15.76, SD = 0.84) underwent the retest of the MCQ-Af after 2 weeks (3 weeks for 1 class of 15, average 2.07 weeks), the same interval as used in the English version of Cartwright-Hatton and al. [6]. Pearson’s correlations in our sample were similar to the English version (*r* between .63 and .77, *p* < .001) with the exception of the MCneg factor’s correlation being much higher in our analysis (.75 versus .24), therefore implying an overall acceptable reliability. We calculated the confidence interval of the correlations for a type I error of .10 and counted the number of individuals included in the interval. The percentage of individuals having scored in that confidence interval ranges from 62.25% to 81.25% (Table 3).

### Relationship between metacognition and anxiety

The analysis of the links between metacognition and anxiety are based on a sample of 197 adolescents (97 boys, 100 girls, between 13 and 17, mean age 15.28, SD = 1.21).

We analyzed the associations using both the MCQ-Af with all 30 items, for comparison with Cartwright-Hatton’s and al. results, and the corrected MCQ-Af27 (minus item 2, 12 and 14). All factor scores of the MCQ-Af and MCQ-Af27 were normally distributed in this sample of 197. Anxiety total scores of the R-CMAS also had a normal distribution and were congruous to the norms. The differences of means of the MCQ-Af scores with the original sample of 214 were non-significant (Table B in S1 Appendix).

All factors of the MCQ-Af27 and total score were positively and significantly correlated with the R-CMAS total score. The weakest correlation *r* = .16, *p* = .029 concerned factor Confidence. The strongest correlations were *r* = .56, *p* < .001 between the 2 total scores and *r* = .56, *p* < .001 between the total score of R-CMAS and factor MCneg (Table C in S1 Appendix). The power of Pearson correlation *r* = .20 for a sample of 197 was 83.2%, maximum power attained with *r* = .35.

Simple linear regressions of the scores of each factor of the MCQ-Af27 were analyzed in relation with the total score of R-CMAS. The results showed that the 5 factors and total score of the MCQ-Af27 had each a positive and significant effect, factor MCneg explaining the most variance along with the MCQ-Af total score, factors Conscience and Confidence having a relatively week influence. When all 5 factors are included in a stepwise regression, Control and Conscience did not bring any significant variance to the model (Table 4).

**Table 4:** Simple and stepwise linear regressions of the 5 factors of the MCQ-Af27 on anxiety scores, N = 197.

	Simple Regression				Stepwise Regression			
	b	*R^2^(%)	t(195)	*p*	b	*R2(%)	t(191)	*p*
MCneg-Q2	.56	30.84%	9.40	< .001	.49	31.19%	7.44	< .001
MCpos	.35	11.65%	5.18	< .001	.13	2.56%	1.93	.007
Confidence-Q14	.16	1.91%	2.20	.029	.12	1.44%	2.06	.039
Control	.31	8.97%	4.51	< .001	.11	1.06%	1.78	.076
Conscience-Q12	.22	4.47%	3.19	.002	-.01	-	-	.869
MCQ-Af27	.56	30.47%	9.32	< .001				

MCpos = positive metacognitive beliefs; MCneg = negative metacognitive beliefs.

*R^2^ = adjusted R^2^

### Influence of age and gender

#### Metacognition

All the analyses were performed on the MCQ-Af27, excluding items 2, 12 and 14. The simple linear regressions showed that taken independently, age had a moderate but significant positive influence on factor Conscience (*F*(1.21) = 6.38, *p* = .012, R^2^ = 2.46%) and total score (*F*(1.21) = 5.81, *p* = .017, R^2^ = 2.21%); gender had a influence on factor MCneg and Control: MCneg: *F*(1.21) = 13.83, *p* < .0001, R^2^ = 5.68%; Control: *F*(1.21) = 5.88, *p* = .016, R^2^ = 2.24%, MCneg and Control being higher in girls.

Furthermore, comparison of means using Student’s *t* test showed that 3 factors, MCneg, MCpos and Conscience, increased significantly and specifically between age 13 and 16 (between 13 and 17 for factor Conscience, differences for the remaining scores between 16 and 17 were non-significant) (Table 5).

**Table 5:** Means of the 5 factors of the MCQ-Af27 between age 13 and 17, N = 214.

	Means 13 to 17	Means at 13	Means at 16	Means at 17	*t*(61)	*t*(64)	*p*
MCpos	11.33	10.22	12.18	11.38	2.05		0.045
MCneg-Q2	10.11	8.67	11.09	9.94	2.42		0.019
Confidence-Q14	8.2	7.11	8.58	8.58	1.54		0.129
Control	11.68	11.5	12.04	11.69	0.60		0.55
Conscience-Q12	12.96	11.56	13.31	13.75		2.3	0.025
MCQ-Af27	54.28	49.06	57.2	55.33	2.72		0.008

MCpos = positive metacognitive beliefs; MCneg = negative metacognitive beliefs. In grey: significant differences.

The stepwise regression showed a participation to total variance of 50.82% for MCneg, 22.61% for Control, 12.98% for MCpos, 6.75% for Confidence and 6.08% for Conscience. When including age and gender, Confidence and Conscience did not participate to the total variance, age accounting for 2.21% and gender being non significant.

#### Metacognition and anxiety

Anxiety scores from the R-CMAS did not vary significantly with age and gender. However, in the stepwise regression including all 5 factors of the MCQ-Af27, as well as age and gender, age added 1.51% and gender 1.58% to the total variance of 37.11%, *F*(6.190) = 20.280, *p* < .001 (Table 6). Age played a particular role in boys (R^2^ = 10.22%, *p* = .002 versus R^2^ = 0.26%, *p* = .621 in girls). Gender differences were significant particularly at age 14, with a contribution to the variance of R^2^ = 15.83%, *p* = .029. All ages confounded, Confidence (along with MCneg and MCpos in a lesser degree) played a more significant role in girls than in boys (R^2^ = 4.12%, *p* = .047 for girls versus R^2^ = 0.78%, *p* = .399 for boys).

**Table 6:** Stepwise linear regression of the 5 factors of the MCQ-Af27 plus age and gender on total anxiety scores, N = 197.

	**Stepwise Regression**			
	**b**	*R^**2**^**(%)**	***t*(190)**	***p***
MCneg-Q2	.55	31.19%	7.96	< .001
MCpos	.11	2.55%	1.93	.007
Gender	-.11	1.58%	-1.77	.029
Age	-.15	1.51%	-2.56	.035
Confidence-Q14	.11	1.30%	2.00	.047
Control	.10	0.8%	1.67	.096
Conscience-Q12	-	-	-	-

MCpos = positive metacognitive beliefs; MCneg = negative metacognitive beliefs. Gender code 0 = boys, gender code 1 = girls.

*R^2^ = adjusted R^2^

## Discussion

Our analyses first showed that the MCQ-Af is a valid and reliable tool, providing the first questionnaire available to assess metacognitions in a French-speaking adolescents sample. The same five factor structure as reported in the English version by Cartwright-Hatton and al. [6] was identified in our study. However, we supported that the best model representing our sample was a bifactor model instead of a higher order model. Secondly, associations between MCQ-Af and R-CMAS scores showed that, consistent with most studies, the factor measuring negative metacognitive beliefs had the strongest influence on anxiety scores. Finally, our analyses showed that MCQ-Af factors and total scores increased with age and that age and gender also influenced the link between MCQ-Af27 and R-CMAS scores. Specifically, we demonstrated that age plays a particular role in boys, thus suggesting differences between younger and older adolescents, as well as between boys and girls. These results are discussed below.

### Validation of the MCQ-A

Our prior path analyses showed that loadings of factors on their items were generally acceptable. However, three items, item 2, 12 and 14, had non-significant loadings and were problematic for our further confirmatory analyses. Item 2 is part of the negative metacognitive beliefs factor (MCneg) and relates to meta-worries and the danger of worrying. It is worth noting that other validation studies of the MCQ-A in other languages have also encountered the same problem particularly with item 2 (i.e. in a Dutch sample [14]). Item 12 relates to factor Conscience or thought monitoring, concepts that might have been difficult to apprehend, particularly in younger adolescents. As for item 14, it contains a time appreciation e.g. « at times », and a heterogeneous interpretation from our young population may underlie the non-significant result. In view of these results, we suggest that these 3 items should be given particular attention and analyzed with caution if included in the MCQ-Af questionnaire when submitted to other French-speaking samples.

Confirmatory analyses using structured equation modeling were therefore achieved using the corrected questionnaire of 27 items, excluding the problematic items 2, 12 and 14. The second order model, as used in the original English validation [6], was compared with the unidimensional model, the bifactor model and the covariate factor model. The models were then adjusted by allowing 4 cross loadings concerning items 9 and 11, as well as 3 and 23. Of note, item 11 was also allowed to cross-load on the same two factors (MCpos and MCneg) to obtain better fits in a MCQ-30 validation study on an adult population [9]. As for item 3, a study on an adult sample showed that this particular item loaded higher on factor MCneg than on its predicted factor Conscience, the same two factors concerned here [49]. We then examined these adjusted models and noticed a better fit for each model particularly in the bifactor model and the covariate factor model (see Fig 1 and Fig 2). In comparison, even if the covariate factor model seemed to indicate overall better fit indices, the degree of freedom and the PGFI penalizing complexity were higher in this model than in the bifactor model. It seems therefore more adequate to consider the bifactor model as the best fit to our sample. Indeed, the bifactor model has drawn more and more attention amongst researchers and recent studies have discribed their advantages [50]. This model is of particular interest because it indicates that most items *share* their variance between the breadth factor and their own specific factor. This model therefore allows a direct reading of the shared variances (all significant here) between the different factors *and* the breadth factor. Furthermore, the items measuring the factor relating to the negative metacognitive beliefs (MCneg) showed strong loadings with the breadth factor. Indeed, many studies state that this specific factor is regularly the most influential and our results illustrate that fact. In summary, the above points might reinforce the relevance of considering a breadth factor rather than a higher order factor. Moreover, it supports the fact that each factor can be considered independently, with its proper contribution and estimated variance, allowing therefore a more explicit illustration of the different patterns of relations between each factor in different pathologies [4, 21–24]. Indeed, studies investigating anxiety and depression, for example [23], revealed that negative beliefs about worry was the strongest predictor for both anxiety and depression and that cognitive confidence, beliefs about the need to control thoughts, and cognitive self-consciousness, predicted depression but not anxiety. Others who investigated PTSD [22] concluded on the effect of negative and positive beliefs, and need to control. Others still, examining OCD, found an influence of negative beliefs, control and self-consciousness [14].

Concerning the influence of gender, the negative metacognitive beliefs (MCneg) and Control, reflecting beliefs about the need of thought control and control on worries, seem to be greater in girls than boys. This corroborates studies that showed that girls [17, 28] and women are generally more prone to develop worries and ruminations [51]. As for the influence of age, we found that metacognitive scores were getting higher as adolescents grow older, particularly in boys between 13 and 16. Comparatively, the study of Cartwright-Hatton and al. [6] did not account for any significant effect, as well as Esbjorn and al., in a sample of children and adolescents between 9 and 17 tested on the metacognition questionnaire for children (MCQ-C30) [18]. Several studies, even if showing an increase of factors’ scores up until the age of 13, noted no significant results in increase after that age in all metacognitive scores of the MCQ-A [6, 25, 26, 28]. Some authors have suggested that metacognitive beliefs at that age could be compared to metacognitive beliefs in adults [25, 27, 29]. In comparison, the majority of studies analyzing the MCQ in adults aged 18+, no change with age was noticed, the variation of scores of all factors, except Confidence, would rather tend to a *decrease* with age, showing simultaneously that younger participants tend to score significantly higher on all factors [23]. Our results may suggest that age has a significant influence on factors measuring positive and negative metacognitive beliefs, and this particularly between age 13 and 16. One’s ability to monitor one’s thoughts measured by factor Conscience seems to even continue to increase until the age of 17. If longitudinal studies would be needed to confirm these findings, our results are nevertheless in accordance with studies in neuroscience and neuropsychology that have revealed that brain areas and connections underlying metacognition and conscience still develop until the age of 22. Indeed, cerebral white matter connecting cortical frontal and temporal regions and subcortical structures (NAcc, Insula, ACC) implicated in emotional evaluation, control and monitoring [37], increase in volume and functional connectivity until mid-to-late adolescence before showing relative stability [36]. In view of the above observations and our results, it seems that scores of cognitive beliefs and processes could still mature in children and adolescents till at least 17 and then decrease during adulthood. This trajectory could therefore be considered as typical in adolescents. Indeed, studies that investigated this same development period also found an increase in worries and anxiety [52], manifestations that are strongly associated with negative metacognitive beliefs, as we will discuss below.

### Metacognition and anxiety

Results of stepwise regression in our study reveal that factor MCneg contributed to the majority of the variance of the link with anxiety scores, along with factor MCpos, and factor Confidence in a lesser degree. Our findings are congruent with numerous studies [4, 21–24] that report positive significant correlations between anxiety scores and all 5 factors and total scores of the MCQ but especially with factor MCneg. Furthermore, some studies have also shown that this factor (MCneg) is the best predictor of anxiety disorders [23]. Those negative beliefs about worries, being considered as dangerous and uncontrollable, are at the center of the Wells’ GAD metacognitive model and are therefore an important measure of possible vulnerabilities or risk factors of anxiety disorders in adolescents.

Concerning age and gender, anxiety scores did not significantly vary in our non-clinical sample. However, in the stepwise regression of metacognition measures on anxiety scores including these two factors, age and gender had a modest but however significant influence on the total variance. It is worth noting that our results indicate a negative influence of age. This would mean that the older the adolescent, the lower are their anxiety scores, but the higher are metacognitive negative and positive beliefs and non-confidence in one’s own memory. This seems to be an interesting point leaving some open questions about what particular component (also possibly age and gender dependent) could decrease the strength of the association between metacognitive beliefs and anxiety manifestations (i.e. cognitive capacities, flexibility, resilience). Indeed, increase of cognitive capabilities and/or flexibility occurring during adolescence could lead to a better control of metacognitive processes like the need to control thoughts, confidence in one’s memory and monitoring of thoughts [28, 53, 54] and could moderate anxiety manifestations activated in part by metacognitive beliefs and related processes. This is corroborated by research in neuroscience suggesting that higher anxiety in children is characterized by both delayed expansion of regions in the prefrontal cortex and an altered trajectory of global cortical thinning [55]. Furthermore, studies showing that grey matter and white matter volumes have different trajectories in males and females and suggesting a more rapid development of the female brain [38], could also partly explain our results indicating that age is particularly relevant in boys and that girls show lower anxiety scores even with relatively higher negative metacognitive beliefs and need to control.

### Limits and future perspectives

The major limitation of our study examining the links between age, metacognition and anxiety is the cross-sectional design. Longitudinal data would permit to measure the intra-individual development of these different factors in time. It is indeed difficult to draw general conclusions on the increase of metacognitive beliefs with this design, particularly when dealing with young adolescents still in the process of cognitive and psychological development.

Concerning the MCQ-A, the influence of the five specific factors on different psychopathology seem to show different patterns, suggesting that other studies including clinical samples would be necessary notably among adolescents. Indeed, the majority of studies is addressing adults and already shows heterogeneous results at ages over 18, as well as amongst the same syndromes. Convergent and discriminative validity processed by these studies might also lead to different results depending of sample number and characteristics, indicating that the MCQ is possibly very sensitive to the specificities of the analyzed population. Furthermore, in most studies clinical samples are usually small and present quite an extensive heterogeneity and comorbidity. In addition, longitudinal studies a still rare in all age groups but particularly in adolescence. Though it would be very important to detect as early as possible risk factors such as dysfunctional metacognitive beliefs and anxiety symptoms that could lead to psychopathologies, and this especially in a critical developmental period.

## Conclusion

In summary, our result concerning the French version of the MCQ-Af obtained in a sample of 214 adolescents aged between 13 and 17, show the best fit for a bifactor model, allowing to consider the five factors independently as well as a general breadth factor. Quality of psychometric properties is satisfactory to good, thus confirming the validation of the scores’ interpretation of the MCQ-Af in a French-speaking population of adolescents from Switzerland. However, items 2, 12 and 14 excluded from our analyses, should be considered with caution. We have been able to show a modest, nevertheless significant, influence by age and gender inside the dimensions of the MCQ-Af and also in their links with anxiety scores. Our general analyses of these links are in adequacy with other studies showing the highest effect from the negative metacognitive beliefs on anxiety scores, along with positive metacognitive beliefs, non-confidence in one’s own memory and in a lesser extend with need of thought control. Future studies, particularly concerning this age group, should investigate longitudinal data from non-clinical as well as clinical samples, to further illustrate the relations between metacognitions, anxiety, age and gender, and possibly help to detect and understand, as early as possible and at a young age, risk factors such as dysfunctional metacognitive beliefs and anxiety symptoms that could lead to psychopathologies.

## Supporting information

S1 Appendix MCQ-Af means and correlations. (PDF)
