# Supplementary material for: Validation of the French version of the « Meta-Cognition Questionnaire » for adolescents (MCQ-Af): Evolution of metacognitive beliefs with age and their links with anxiety during adolescence
Source: PLoS One. 2020 Mar 11;15(3):e0230171. doi: 10.1371/journal.pone.0230171 (PMC7065794; doi:10.1371/journal.pone.0230171)
Supplement: S1 Appendix — (PDF) [file pone.0230171.s001.pdf]

S1 Appendix. MCQ-Af means and correlations

Table A. Correlations between the 5 factors of the MCQ-Af27 and the MCQ-Af27 total score; N = 214.

	MCneg - Q2	Confidence - Q12	Control	Conscience - Q14	MCQ-Af27
Mcpos	0.41***	0.14*	0.33***	0.34***	0.71***
MCneg - Q2		0.23**	0.32***	0.32***	0.71***
Confidence - Q12			0.19**	0.017	0.48***
Control				0.32***	0.68***
Conscience - Q14					0.62***

In grey: non-significant correlation.
*** p < .001 ** p < .01 * p < .05

Table B. Comparison of means and standard deviations (SD) of the 5 factors of the MCQ-Af30 and MCQ-Af30 total score.

			**C-H**	**C-H**	**C-H**
	**N = 214**	**N = 197**	**N = 166**	**non-clinical *n* = 11**	**clinical *n* = 11**
MCQ-Af30	61.48 (12.01)	61.93 (11.93)	58.5 (15.0)	54.09 (17.90)	69.82 (12.02)
MCpos	11.29 (3.89)	11.37 (3.82)	10.7 (4.5)	10.72 (5.92)	9.73 (3.64)
MCneg	12.03 (3.81)	12.40 (4.01)	12.4 (4.8)	10.18 (3.92)	18.45 (2.54)
Confidence	10.73 (3.83)	10.64 (3.61)	10.3 (3.7)	8.10 (2.17)	11.82 (4.42)
Control	11.66 (3.67)	11.87 (3.73)	11.5 (3.6)	11.10 (3.86)	14.91 (3.48)
Conscience	15.75 (3.72)	15.65 (3.66)	13.9 (4.0)	14.00 (4.69)	15.36 (3.61)

Means in Cartwright-Hatton and al. (C-H) total sample N = 166, non-clinical and clinical subsamples *n* = 11 and our samples of N = 214 and N = 197. In grey significant differences in Cartwright-Hatton and al. (2004).

Table C. Correlations between the 5 factors of the MCQ-Af27, MCQ-Af27 total score and anxiety score; N = 197.

							**C-H**
				**N = 197**			**N = 143**
	Mcneg - Q2	Control	Confidence - Q12	Conscience - Q14	MCQ-Af27	RCMAS	RCMAS
MCpos	0.36***	0.33***	0.15*	0.34***	0.68***	0.35***	0.32***
Mcneg - Q2		0.31***	0.02	0.30***	0.70***	0.56***	0.67***
Control			0.06	0.30***	0.69***	0.31***	0.42***
Confidence - Q12				0.08	0.20**	0.16*	0.47***
Conscience - Q14					0.61***	0.22**	0.35***
MCQ-Af27						0.56***	0.62***

Correlations in Cartwright-Hatton and al. (C-H) N = 143 and our samples of N = 197. MCpos = positive metacognitive beliefs; MCneg = negative metacognitive beliefs. In grey: non significant correlations.
*** p < .001 ** p < .01 * p < 0.05
